# The Efficiency of Phosphate Removal via Shallow Wastewater Injection into a Saline Carbonate Aquifer

**DOI:** 10.1021/acsestwater.4c00407

**Published:** 2024-07-26

**Authors:** Kate Meyers, Megan Martin, Lee R. Kump, Miquela Ingalls

**Affiliations:** Department of Geosciences, The Pennsylvania State University, University Park, Pennsylvania 16802, United States

**Keywords:** wastewater remediation, phosphate, nutrients, mineralization, injection well

## Abstract

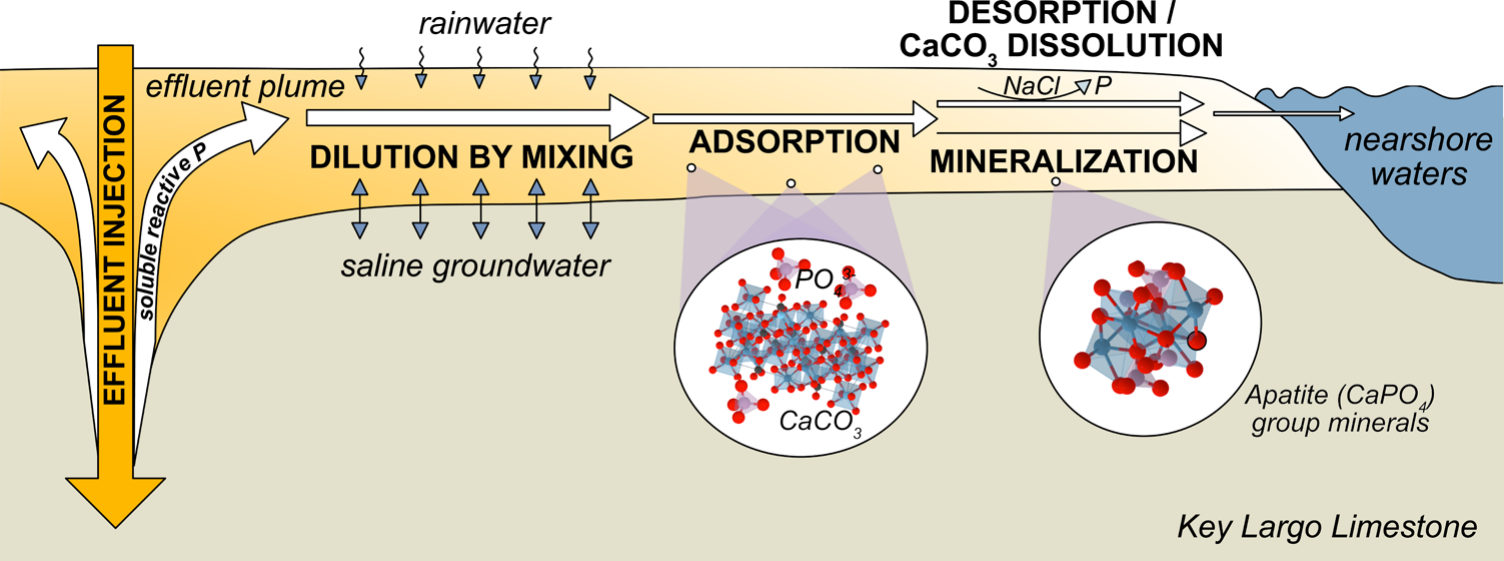

Wastewater-derived phosphate contributes to eutrophication if the phosphate is not efficiently removed before it is discharged to surface waters. In the Florida Keys (USA), shallow injection of treated wastewater into saline limestone aquifers is a common mode of wastewater disposal. We assessed the possibility of efficient and permanent phosphate removal following injection at a wastewater treatment facility in Marathon, Florida. The concentrations of nutrients, dissolved ions, and anthropogenic compounds in groundwater and nearshore waters were monitored over two years, as was the progression of a patch of fluorescent dye emplaced by the wastewater injection well. The density contrast between the wastewater effluent and saline groundwater caused the effluent plume to buoy to the shallow subsurface near the injection well. Soluble reactive phosphorus (SRP) and sucralose were both detected in nearshore waters, indicating incomplete removal of contaminants. However, ∼75% of the SRP is removed from the plume in the first 10 days of transit by adsorption followed by a slower removal mechanism, bringing the P removal efficiency above 90%. A positive relationship between excess calcium and phosphate removal efficiency, together with high levels of calcium phosphate mineral supersaturation, supports calcite dissolution followed by calcium phosphate mineralization as this slower removal process.

## Abstract 2

Phosphate is efficiently removed from saline groundwater after injection of treated wastewater effluent by adsorption and calcium phosphate mineralization.

## 1 Introduction

Anthropogenic nutrient loading in coastal environments can be detrimental to the health of coastal ecosystems. Municipal wastewater, in particular, has been the subject of debate and legal action in coastal communities in the United States in recent years.^1,2^ Wastewater disposal potentially contributes anthropogenic phosphate and nitrogen to nearshore waters, even with advanced wastewater treatment. Phosphate is essential for primary producers such as phytoplankton,^3^ but too much phosphate can cause eutrophication and harmful algal blooms.^4,5^

The protections that the Clean Water Act has in place for point source discharge of anthropogenic contaminants into navigable waters were upheld in the 2020 Supreme Court decision on *County of Maui v. Hawaii Wildlife Fund*. Specifically, this decision held that a permit is required “if the addition of pollutants through groundwater is the functional equivalent of a direct discharge from the point source”.^1^ Examples of point sources are industrial pipes, residential septic tanks, and shallow wells used to inject municipal wastewater effluent into the ground. However, whether a nutrient or contaminant load can be considered the functional equivalent of direct discharge depends on an array of factors: e.g., path length and substrate of flow path from point source to nearshore waters, hydrology of the groundwater system, bedrock geology and reactivity, and biogeochemical processes. In the case of discharged phosphate, a potentially efficient mode of subsequent removal is by chemical adsorption onto limestone mineral surfaces encountered in the flowpath. We evaluated the efficacy of anthropogenic phosphate removal by adsorption onto limestone surfaces following the shallow injection of wastewater effluent. The goals of this study were to determine the fate and transport of phosphorus in the groundwater and determine the potential for permanent reactive phosphate removal by calcium phosphate mineralization.

### 1.1 Wastewater Phosphate Remediation in the Florida Keys

The oligotrophic waters of the Florida Keys National Marine Sanctuary (FKNMS, 9800 km^2^ of protected marine ecosystems) are sensitive to anthropogenic inputs of phosphate, the limiting nutrient in Florida Bay.^6^ The regulation of and improvements to wastewater treatment and disposal have been the subject of intense scrutiny in South Florida for decades (e.g., ref (7)). Although advanced wastewater treatment (AWT) and shallow (and in some cases, deep) injection have largely replaced the cesspits, direct outflows and secondary treatment, shallow injection wells of the past, water quality in the nearshore regions of the Florida Keys remains impacted. Understanding the chemical mechanisms by which nutrients such as phosphate are removed from wastewater effluent and their limits is of utmost importance.

Many wastewater treatment facilities in the FKNMS employ shallow injection of treated effluent through a cased well <30 m into the carbonate bedrock. In theory, dissolved inorganic phosphate that remains in the effluent after leaving the wastewater treatment facility will be adsorbed onto the limestone surfaces in the travel path between the injection well and other water sources or until the effluent is sufficiently diluted by groundwater. However, laboratory and field studies have found that the sorption mechanism is weakened in seawater because of competing ion concentrations, and may result in P desorption.^8−11^ Despite these potential drawbacks, past field studies on shallow injection wells have observed the efficient uptake of wastewater-derived phosphorus through its interaction with Key Largo Limestone (KLL), the porous bedrock beneath the middle Florida Keys.^12−14^ Corbett and colleagues^12^ found that 95% of phosphate was removed from the effluent plume prior to emergence in nearby canals. However, this study was performed at an injection well that disposed of a factor of 1000 less wastewater than the maximum daily rates observed at current, more centralized treatment facilities in the middle Keys. Dillon^13^ found that most of the phosphate disposed of at such a facility, Key Colony Beach in the middle Florida Keys, was removed from the effluent plume prior to emergence in nearshore waters. However, monitoring wells 78 and 160 m from the injection site that measured below the method detection limit at the beginning of the study later yielded phosphate concentrations of ca. 10 and 8 μM, indicating the possible progression of a phosphate-limestone exchange-equilibrated front. Thus, although phosphorus from the effluent plume had not yet reached the nearshore waters, significantly elevated P levels in the distal groundwater wells indicated that P was not effectively removed from groundwater. Carbonate lattices in the flow path may have become saturated with phosphate after continuous exposure to the phosphate-rich effluent plume, which reduces the P sorption capacity. The findings of Dillon^13^ highlight the need to understand the limits of phosphate uptake via adsorption and the permanence of this phosphorus removal mechanism. Here, we characterize the travel time, path, and geometry of the treated effluent plume, quantify the capacity of phosphate adsorption onto limestone bedrock surfaces in the saline aquifer, and evaluate the phosphate load that emerges into nearshore waters.

### 1.2 Site Description

**Figure 1:**
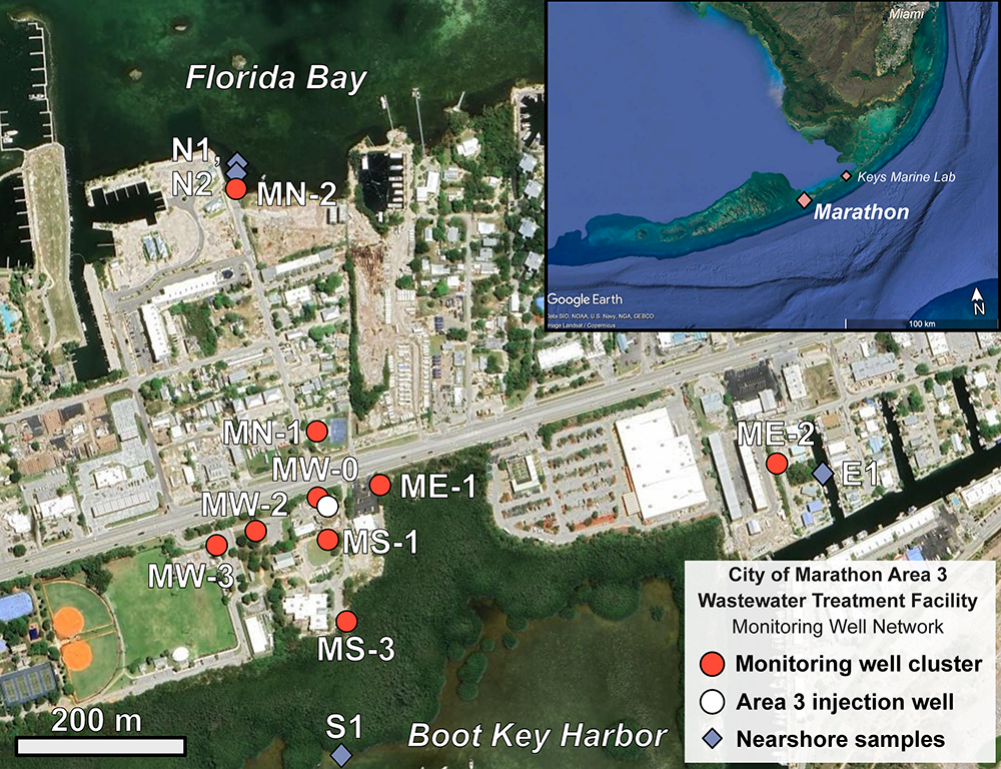
Location of field study in Marathon, FL. Boot Key Harbor resides to the south, and Florida Bay resides to the north of the Area 3 Treatment Facility.

We focus this study on the groundwater and nearshore waters at the Area 3 Wastewater Treatment facility in Marathon, FL in the middle Florida Keys (Figure 1; Text S1). The bedrock of the middle Florida Keys is composed of Key Largo Limestone, a skeletal packstone to wackestone with macroscopic vuggy porosity that was deposited during the Pleistocene under higher sea level conditions and has subsequently undergone meteoric diagenesis,^15,16^ including considerable conversion of primary aragonite to secondary calcite. Primary and secondary porosity of >45% allows for rapid flow in the subsurface^16^ with a hydraulic conductivity of ∼1400 m/day.^17^ Key Largo Limestone is the sole geological reservoir and conduit through which the injected wastewater migrates beneath Marathon before reaching Florida Bay and the Atlantic Ocean. Past studies on smaller treatment facilities in the middle Florida Keys have found that wastewater can reach the nearshore waters within days to weeks.^12−14^ The City of Marathon currently uses shallow injection wells to pump treated effluent between 18 and 27 m into KLL. The studied facility is permitted to 1.3 × 10^6^ liters per day (L/d). Average daily flow rates from 2020 to 2023 fell below this limit, ranging from 6.4 to 7.2 × 10^5^ L/d. However, over the same period, the maximum daily flow rates reached 1.4–3.4 × 10^6^ L/d according to publicly available utility data.

## 2 Methodology

### 2.1 Field Methodology

Ten monitoring well clusters were installed in 2021 and 2022 with wells drilled to sampling depths of 3, 6, and 15 m at all sites and 27 m at three sites (Table S1; further details of well construction in Text S2 and Figure S1). Three sampling trips were completed in November 2021, May 2022, and January 2023. Wells were adequately purged by a peristaltic pump prior to sample collection (Text S2). Details of collection of filtered and unfiltered well water samples and field measurements of physicochemical parameters (i.e., temperature, pH, and conductivity) can be found in Text S2. Practical salinity was measured and reported as grams of salt per kilogram of solution (g/kg). Nearshore surface water samples were collected for aqueous geochemical analysis in January 2023. The locations of these grab samples were the nearest navigable waters that the effluent plume could reach to the north (N1, N2), south (S1), and east (E1) of the injection well (Figure 1).

We performed a two-step fluorescein dye tracer study with two injections one month apart (Text S3). Dye was added from an opaque storage tank to the effluent injection port of the City of Marathon Area 3 wastewater treatment facility. The flow rate of effluent at the time of the dye study was 75 L/min. Fluorescence was measured using an AquaFluor hand-held fluorometer calibrated with a blank and 10 and 400 ppb standards. Proximal wells (MW-0) were purged and measured for fluorescence 6 times over the course of 3 h after the initial dye injection to observe the arrival of the dye pulse. Sampling occurred hourly to daily at the other nearby well clusters for the first 10 days. After this point, sampling slowed to 3–4 days per week, focusing on the central wells with occasional sampling of the outermost well locations (ME-2, MN-2, MW-3, and MS-2). As the dye arrived at different well locations, sampling became more frequent as needed and possible to best capture the peak arrival (Table S2).

### 2.2 Laboratory Methodology

Soluble reactive phosphorus (SRP), total phosphorus (TP), total nitrogen (TN), dissolved organic carbon (DOC), and concentrations of anthropogenic organic compounds were analyzed by following standard EPA methods (Text S2). Briefly, concentrations of acetaminophen, sucralose, and carbamazepine were analyzed by high-performance liquid chromatography tandem mass spectrometry (HPLC-MS-MS) at the Florida Department of Environmental Protection (Tallahassee, FL) following standard EPA protocol 8321B. Particular attention was given to sucralose because it is an artificial sweetener present in the wastewater effluent, not removed by advanced wastewater treatment, and only minimally breaks down in the environment. Therefore, sucralose is a prime conservative tracer of wastewater effluent and can be used to distinguish rainwater and wastewater end members that have similar impacts on salinity. Anions were measured via a Dionex ICS 2100 Ion Chromatography System (IC), and cations were measured via Thermo iCAP 7400 Inductively Coupled Plasma Emission Spectrometry (ICP-AES). Sample preparation, handling, and analytical procedures can be found in Text S2. Methods detection limits (MDL) and practical quantification limits (PQL) can be found in the appropriate Supporting Information tables for each analysis.

## 3 Results

### 3.1 Aqueous Geochemical Analyses of Salinity, and Concentrations of Dissolved Ions, Pharmaceuticals, and Nutrients in Groundwater

**Figure 2:**
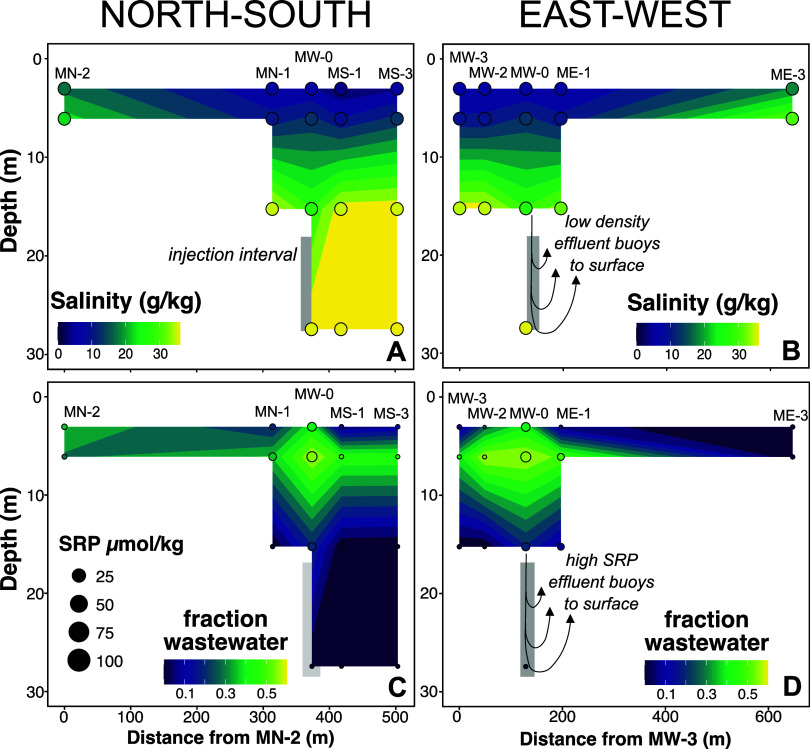
North–south (A, C) and east–west (B, D) cross section maps contoured by salinity reported as grams of salts per kilogram of solution (A, B) and fraction of wastewater (C, D) in well samples from January 2023. SRP concentrations are depicted by the size of the circles in panels C and D. The structure of the subsurface salinity distribution was comparable each sampling season. Determination of the fraction of wastewater in the well samples is presented in Section 4.2.

The salinity of samples collected from the monitoring well network ranged from 4 to 36 (Table S6). In this system, three sources of water impact the salinity values measured: saline groundwater at a salinity of ∼35 g of salts per kilogram solution, rainwater assumed to have a salinity of 0 g/kg, and effluent wastewater with a salinity that ranged from 1 to 2 g/kg during the four sampling periods. A fourth source of water, stormwater that is either allowed to infiltrate in shallow perforated wells or, in some cases, is gravity injected into the subsurface between 18 and 27 m through distributed wells, is not considered in this analysis. The 3 m wells all had salinity values that fell below 10 g/kg except for the outermost wells MN-2 and ME-2 that ranged from 16 to 18 g/kg (Figure 2). Salinity at the 6 m depth wells ranged from 8 to 14 g/kg for the inner cluster of wells and ranged from 17 to 31 g/kg at MN-2 and ME-2. Salinity at the 15 m depth wells ranged from 30 to 35 g/kg at all well clusters except for MW-0, located within 4 m of the wastewater injection well with salinity values between 20 and 27 g/kg. All 27 m wells, excluding MW-0 with a salinity of ∼29 g/kg, had a salinity that ranged from 31 to 35 g/kg. Dissolved ions known to be conservative in seawater are strongly positively correlated with chloride concentration (Figure S2; Table S5).

**Figure 3:**
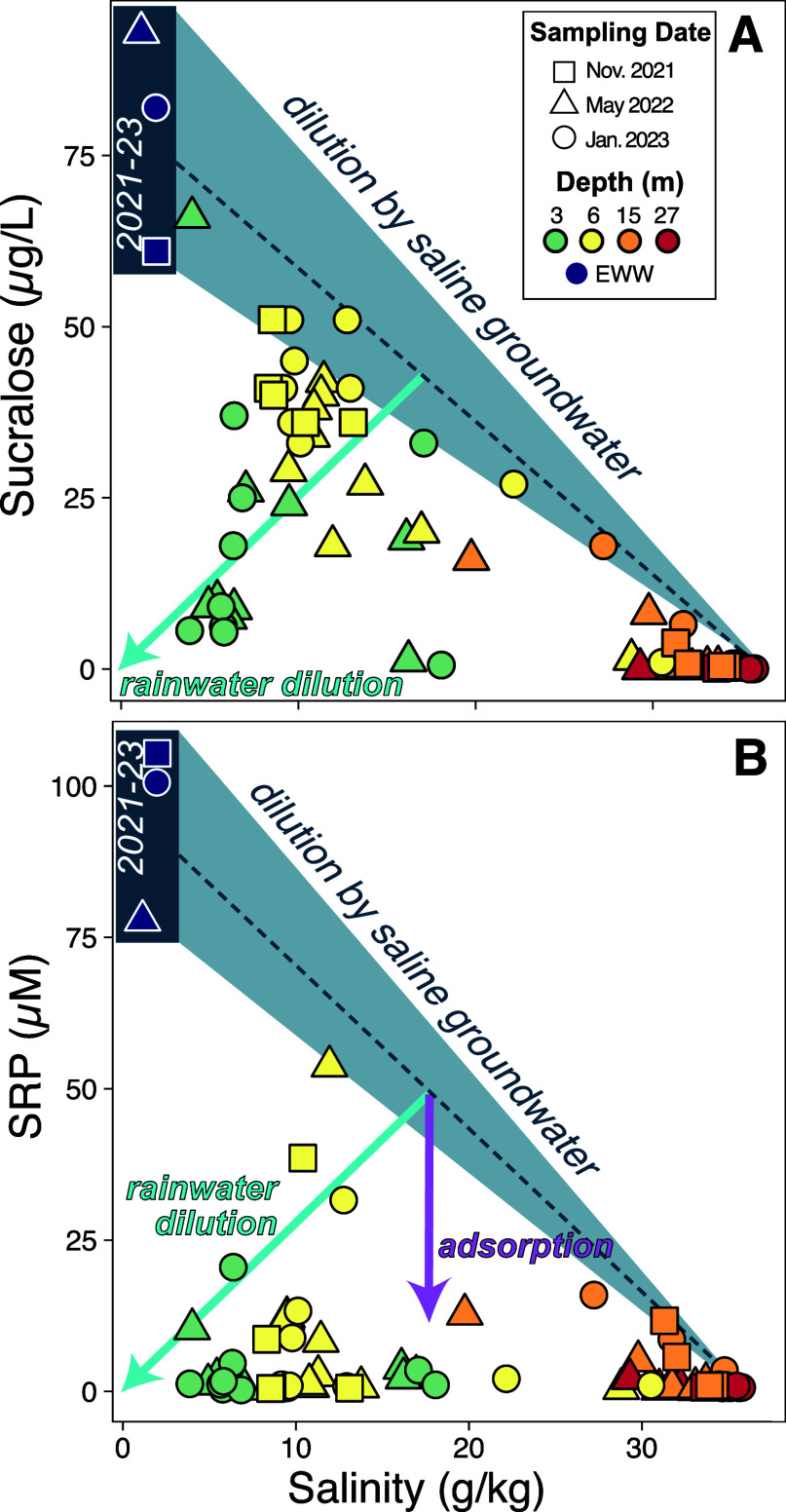
Scatter plots of sucralose concentrations versus salinity and SRP vs salinity. The blue envelope captures the range of sucralose and SRP concentrations expected by dilution of EWW by groundwater with the blue arrow depicting the direction mixing with rainwater would have on the sucralose, SRP, and salinity of each well location. The pink arrow denotes the directionality of the impact SRP adsorption onto the limestone surfaces would have on SRP concentrations in well water.

Sucralose, an anthropogenic compound found in wastewater but absent in groundwater, was the only measured organic compound that fell above detection limits consistently in the effluent and groundwater samples (Figure 3A; Table S4). The concentration of sucralose in the effluent wastewater ranged from 61 to 93 μg/L. Samples from the 3 and 6 m wells yielded the highest sucralose concentrations, averaging 17 and 34 μg/L, respectively. Sucralose was found above detection limits in all 15 m wells, with an average of 2.8 μg/L but with most wells falling below 1 μg/L. Sucralose was not found above the detection limit at any of the 27 m well locations.

SRP followed trends similar to those of sucralose with the highest concentrations of SRP at 6 m, followed by 3 m, then 15 and 27 m. However, nearly all wells were depleted in SRP relative to the effluent wastewater, which averaged 95 μM (Figures 2C,D and 3B). The 6 m wells averaged 7.7 μM followed by the 3 and 15 m wells with averages of 4.1 and 3.8, respectively. The 27 m wells averaged an SRP concentration of 0.78 μM.

Sucralose concentrations measured above the detection limit at all four nearshore localities (Figure 1), ranging from 70 to 370 ng/L (Table S4; Text S4). SRP concentrations were also found above detection limits at E1 (0.12 μM) and S1 (0.01 μM). Mat-forming macroalgae were identified at the three locations seen in the mangrove roots at E1 and S1 and on rock surfaces close to shore near N1.

### 3.2 Dye Tracer Study

The peak arrival of the dye patch was detected at the most proximal wells (MW-0) within 24 (3 and 6 m), 48 (15 m), and 72 (27 m) hours after injection (Figures S3 and S4; Table S2). Peak arrivals were detected at ME-1 15 m and MN-1 6 m approximately one and 2 weeks after injection, respectively, although a higher concentration of the dye was detected in the later peak at MN-1 6 m than the earliest peak arrival at ME-1 15 m by a factor of ∼5 (Figure S4). These early results indicated that the plume’s primary flow path was to the north and east of the injection well with a greater flow to the north. Additionally, the higher concentration and earlier peak arrival at ME-1 15 m than at 3 or 6 m indicated a preferred travel path deeper in the subsurface between the injection well and ME-1 site. More dilute peak arrivals were detected at MN-2 3 m, MW-2 6 m, and MS-3 6 m (Figure S4; Text S4).

Velocity estimates based on peak arrival times and map distances to the wells range from 10 m/day to the east and 5 m/day to the north, to less than or equal to 1 m/day to the south and west (Table S7).

## 4 Discussion

### 4.1 SRP Relationship to Conservative Mixing

The effluent wastewater plume is progressively more diluted by mixing with ambient groundwater the further the plume migrates from the point source injection well. Thus, to assess how much of the reduction in SRP concentration can be attributed to the phosphate–carbonate interaction rather than simple dilution, we compared concentrations of SRP with sucralose and salinity (Figure 3). Sucralose is a useful conservative tracer of wastewater effluent because it has negligible bioaccumulation and sorption potential and is relatively stable under the conditions of the subsurface.^18^

The salinity of shallow groundwater, sampled at depths of 3 and 6 m, consistently fell below 16, indicating mixing of the unmodified saline groundwater and at least one freshwater source. In this system, the two endmembers that would decrease the salinity of the well water are rainwater and wastewater effluent with salinities of ∼0 and ∼2 g/kg, respectively (recall that we are unable to assess the importance of infiltrated and injected stormwater). Except at MW-0 and ME-1, all 15 m wells yielded salinity >30 g/kg, indicating the water at these depths is primarily saline groundwater with minor wastewater influence. At 27 m, all wells outside of the treatment facility yielded a salinity of 31–35 g/kg, indicating that water at this depth is composed dominantly of saline groundwater.

We performed a series of calculations to determine the fractional contribution of the three end-member wastewater (*w*), rainwater (*r*), and the unmodified, saline groundwater (*g*):
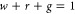
1

The measured concentration of sucralose at each monitoring well, [suc]_s_, reflects the relative contributions from sucralose present in the wastewater effluent, rainwater, and ambient groundwater:
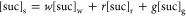
2

Sucralose is an anthropogenic compound and, thus, should not be present in appreciable quantity in rainwater or the saline groundwater (i.e., [suc]_r_ and [suc]_g_ both = 0). As such, this equation can be simplified to determine the fraction of wastewater present at each monitoring well.
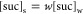
3
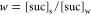
4

The salinity data can then be used to determine the fraction of groundwater present at each monitoring well as follows: where *S*_s_ is the measured salinity of the sample, *w* is the fraction of groundwater solved for with eq 4, *S*_w_ is the salinity of the wastewater, *S*_r_ is the salinity of rainwater, and *S*_g_ is the salinity of the saline groundwater.
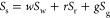
5

*S*_r_ is assumed to be 0 g/kg. With this assumption, eq 5 can be rearranged to solve for the groundwater fraction of each well sample:
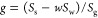
6

Finally, after the ambient groundwater and wastewater fractions of each sample are determined, the rainwater fraction can be determined using eq 1.

**Figure 4:**
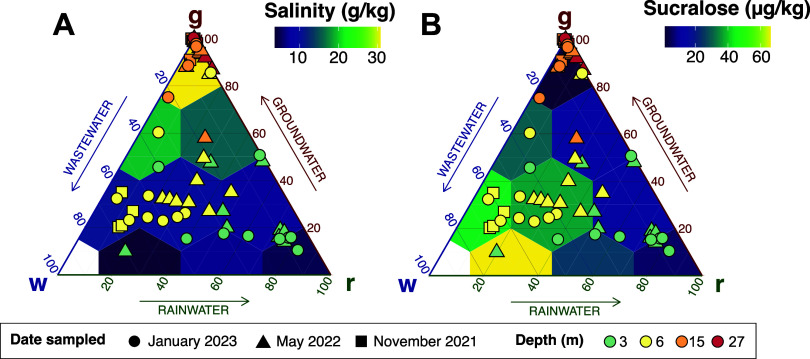
Ternary diagrams depict the mass fraction of rainwater, wastewater, and saline groundwater in each sample. A: Highlighted areas represent a greater influence of each end-member; green: rainwater, blue: wastewater, and red: saline groundwater. B: Background filled in by average salinities or C: sucralose concentrations of samples that plot within hexagonal bins.

Most samples collected from 6 m contained more than 40% wastewater, supporting our initial observation that wastewater primarily flows through the subsurface around a depth of 6 m (Figures 2 and 4; Table S7). Most samples from 3 m, despite yielding similar salinities as 6 m samples, contain <40% wastewater and >50% rainwater. The 15 and 27 m wells were tightly clustered at >80% saline groundwater. The major outlier in the deeper wells was ME-1 15 m, containing higher concentrations of wastewater. This supports the findings of the fluorescent dye tracer study that there was a preferential deeper subsurface flow path from the injection facility to ME-1 at 15 m.

### 4.2 Phosphorus Uptake

To quantify the amount of anthropogenic phosphate removed from the effluent plume along its flow path, we first determined the expected concentration of SRP by multiplying the concentration of SRP in the wastewater by the fraction of wastewater present at the individual monitoring wells (eq 7). This represents the concentration of SRP expected in a well sample if there were no removal of phosphorus along the flowpath:
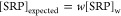
7

The actual concentration of SRP present in the sample ([SRP]_s_) is then subtracted from this expected value to determine the amount of SRP that is removed.
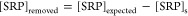
8

**Figure 5:**
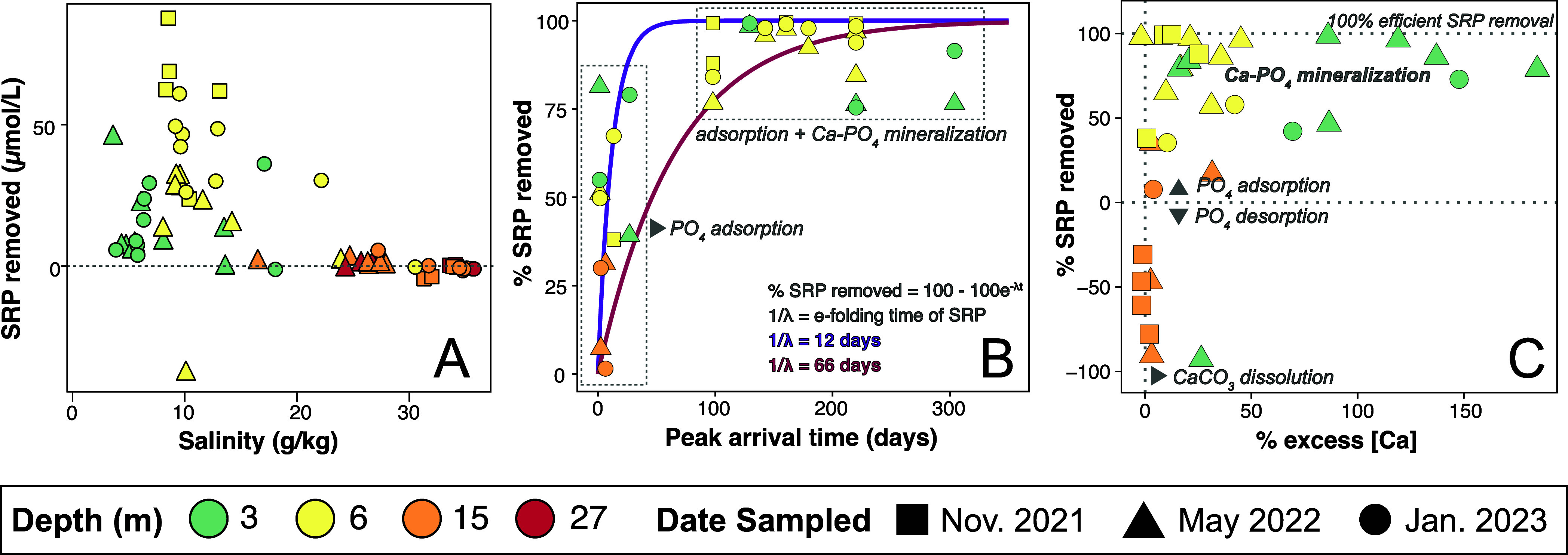
(A) Soluble reactive phosphorus (SRP) removed from the effluent plume across well locations based on estimated SRP values determined in eqs 7 and 8. (B) Percent of SRP removed relative to expected SRP based on *w*. Percent of SRP removed along the flow path demonstrates that the most distal wells experience the greatest SRP removal efficiency. (C) Percentage of soluble phosphate removed relative to percentage of excess calcium in well samples. Values falling above 0% SRP removed indicate higher rates of SRP removal, and values below 0% indicate P desorption. The percentage of SRP removed approaches 100% efficiency in samples with more than 100% excess calcium (see calculations in Section 4.4) likely due to calcium carbonate dissolution and potentially precipitation as Ca-PO_4_ minerals. Samples with % SRP removed < −100%, and the 27 m well depth samples were excluded from this panel.

Positive values of [SRP]_removed_ indicate adsorption or precipitation, while negative values indicate the apparent release of phosphorus back into the groundwaters (Figure 5; Table S7). We also calculated the percentage of SRP removed relative to the expected SRP concentration to normalize for variability in [SRP]_expected_ with proximity to the injection well and flow path:
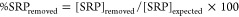
9

A %SRP_removed_ value of 0 would indicate that the [SRP] at that location is equal to the [SRP] predicted based on the fraction of wastewater in the sample; i.e., [SRP] is determined entirely by mixing of the three-component system. A %SRP_removed_ of >0 indicates that [SRP] is lower than the expected concentration by mixing alone.

In general, the wells with the lowest salinities show the largest amount (Figure 5A, Figure S5A) of SRP removed. Ignoring for the moment wells with negative %SRP_removed_, wells with later peak arrival times for the dye tracer showed the highest %SRP_removed_ (Figure 5B). Assuming first-order kinetics controls SRP removal, a best fit line to these data suggests an 66-day e-folding time for SRP removal (Figure S5C). In detail, it appears that a much shorter (12-day) e-folding time fits the data better in the first 50 days. The wells that have lower % SRP removal beyond 200 days include MW-3 3m, which may be receiving fertilizer applications (adjacent to a community athletic field) and MN-2 20m, adjacent to Florida Bay and subject to significant salinity fluctuations from one sampling period to the next, which may lead to SRP desorption.

In fact, several wells yielded negative %SRP_removed_ values (Figure 5C; Table S7). A %SRP_removed_ value below 0 could indicate desorption of phosphate from the KLL surfaces or dissolution of the KLL, which would then release adsorbed phosphate into the groundwater, causing a local increase in SRP. The former can occur when loosely adsorbed P interacts with saline groundwater or a seawater incursion. Based on past field and laboratory studies, this saline water will increase the rate of phosphate desorption and increase the amount of SRP in the groundwater.^10,11,19^ One scenario in which we would expect this to occur is when a larger effluent plume reaches deeper depths during periods of higher effluent discharge and subsequently wanes during periods of lower discharge. Plume contraction and replacement with saline groundwater that follows when injection rates decrease may result in the release of the phosphate adsorbed onto the KLL at these deeper depths. Similarly, when seawater incursions occur, the increase in salinity of the groundwater can promote desorption of loosely adsorbed ions (e.g., refs (9) and (20)).

The SRP removal calculations were based on the average wastewater SRP concentration of the three sampling periods, which ranged from 78 to 105 μM. Because the SRP concentrations of the wastewater effluent vary significantly with time, the actual wastewater SRP concentration may not be well represented by what we use to characterize it, i.e., the average over the three sampling periods. If the initial SRP concentration of the wastewater effluent, which was at the location of the wells with apparent negative SRP removal at the time of sampling, was higher than this average value, our calculated expected value would be lower than actual, resulting in a negative removal concentration. Therefore, the apparent release of SRP at these wells may in fact be an artifact of uncertainty in the initial SRP concentration.

Carbonate minerals have a finite number of lattice sites that phosphate may adsorb to, and thus, over time, the carbonate crystallographic lattice sites may become saturated.^21^ However, our results demonstrate that, even after many years of wastewater injection at this site, rapid and extensive SRP removal is occurring within days and 10s of meters of the site of injection. Thus, the subsurface at this location appears far from saturation with respect to SRP adsorption.

Phosphate is generally considered to be largely removed in the subsurface by adsorption, which, as discussed, is impermanent.^9,19^ Thus, it is vital to examine the mechanisms that could permanently incorporate phosphorus into carbonate minerals for effective, long-term removal of nutrient removal. Previous studies have shown that phosphorus uptake occurs in two steps: a rapid loose adsorption to the carbonate mineral surface followed by a slower removal step.^8,13,22^ One hypothesized mechanism for the latter step is precipitation of a phosphate-containing carbonate mineral.^8,23^ Millero and colleagues^8^ hypothesized that the slower rate of uptake may be attributed to the precipitation of amorphous calcium phosphate that may then transform into a crystalline calcium phosphate phase, such as hydroxyapatite (Ca_5_(PO_4_)_3_OH, or HAP) or carbonate fluorapatite (Ca_5_(PO_4_)_3_F, or CFA). The presence of fluoride in experimental solutions increases phosphate sorption to carbonate, and thus, it has been hypothesized that the crystalline phase could be CFA.^22^ Finally, experimental studies have found that partial dissolution of CaCO_3_ with adsorbed phosphate, which is one possible explanation for elevated [Ca^2+^] in some of the shallow well samples in this study (Figure 5C, Figure S2), promotes mineralization of Ca-PO_4_ solids, which would represent a more permanent reservoir for anthropogenic phosphate.^24^

### 4.3 Mixing Zone Dissolution Associated with Efficient SRP Removal

Mixing of two or more fluids, even if they are saturated with calcite or aragonite but with different salinities, can yield a solution that is undersaturated with calcite because of the nonlinear relationship between mineral solubility and ionic strength (salinity).^25^ Thus, it is possible that mixing of wastewater effluent, rainwater, and saline groundwater leads to a decrease in the carbonate mineral saturation state (Ω = [Ca^2+^][CO_3_^2–^]/*K*_sp_*, where *K*_sp_* is the apparent thermodynamic solubility product for the mineral in question) in the 3–6 m wells with the highest rainwater and wastewater effluent mass fractions (Figure 4). If so, we would expect higher calcium concentrations ([Ca^2+^]_excess_) than those predicted by ternary mixing ([Ca^2+^]_expected_). To test this hypothesis, we calculated [Ca^2+^]_expected_ and [Ca^2+^]_excess_ as follows:
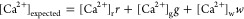
10
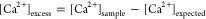
11
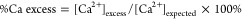
12

We used the same mass fractions as calculated in Section 4.1 and a single value for the Ca concentrations of groundwater and wastewater effluent for all seasons, which were 10.2 mM (January 2023, MS-3 27m) and 1.57 mM (January 2023, EWW), respectively. We did so to account for the travel time in the subsurface and resulting discordance in the composition of EWW at the treatment facility and mixed with groundwater in the distal wells at the time of sampling. We used Ca concentrations of rainwater collected from Site FL11 of the National Atmospheric Deposition Program in the month of our sampling for [Ca^2+^]_r_.^26^ This site is located in Everglades National Park and is the closest NADP precipitation monitoring site to the Florida Keys. However, we note that, although precipitation chemistry at Site FL11 is also likely influenced by sea-salt entrainment, we expect the influence of sea salt on precipitation chemistry in the middle Keys to be larger. This adds a small but nonnegligible uncertainty to our calculations.

**Figure 6:**
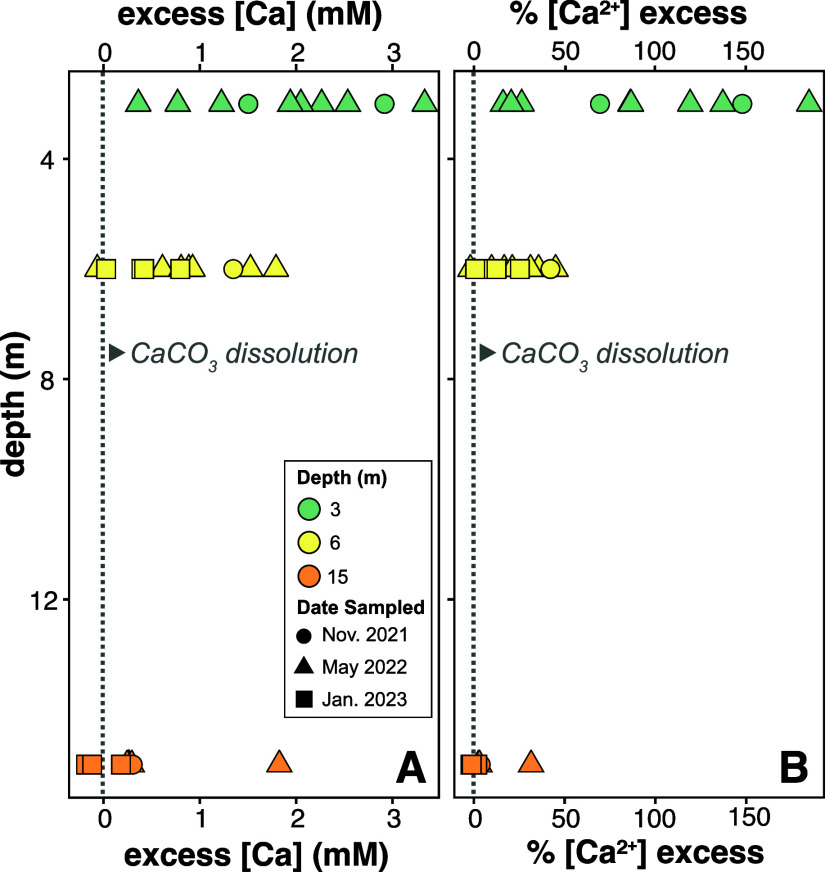
Calcium in excess of expected [Ca^2+^]. [Ca^2+^]_exp_ is calculated by ternary mixing of groundwater, wastewater effluent, and rainwater at each site and depth. (A) Concentration of excess calcium and (B) the percentage of excess calcium relative to expected are highest in the shallowest wells with greatest influence from rainwater mixing.

Using these values, we determined that all of the samples at 3 m and the majority at 6 m depths have excess [Ca^2+^] as compared to the expected [Ca^2+^] from ternary mixing (Figure 6; Table S7). We attribute the excess calcium to dissolution of primary aragonite and/or secondary calcite in the Key Largo Limestone because of undersaturation developed during mixing and the undersaturation of the treated effluent wastewater with respect to aragonite and calcite. Dissolution of calcium carbonate with adsorbed P would release the P back into solution. The fate of the desorbed P determines whether calcite dissolution has detrimental implications for the environment. The question is does the desorbed P remobilize in the subsurface and, ultimately, emerge in surface waters, or is desorbed P sequestered as Ca-PO_4_ minerals?

**Figure 7:**
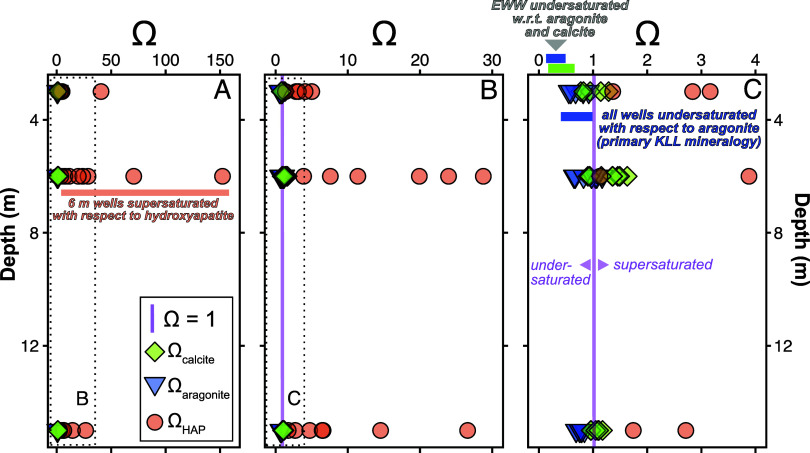
Mineral saturation states (Ω) for calcite, aragonite, and hydroxyapatite. The majority of groundwater samples were strongly supersaturated with respect to hydroxyapatite, modestly supersaturated to undersaturated with respect to calcite, and undersaturated with respect to aragonite. The effluent wastewater was undersaturated, with respect to both calcite and aragonite.

To test whether the excess calcium was likely sourced from dissolution of the Key Largo Limestone and to evaluate the likelihood of remineralization of desorbed P, we calculated mineral saturation states (Ω) for calcite, aragonite, and hydroxyapatite (Figure 7) using the dissolved ion concentrations, pH, and temperature of the well samples and effluent wastewater (Table S9), and the total alkalinity measurements for each well from July 2021. We used the open-source thermodynamic aqueous geochemistry program PHREEQc^27^ using the *phreeqc* package in R (v.3.7.6; ref (28)) and the Lawrence Livermore National Laboratory thermodynamic database (llnl.dat) with the addition of hydroxyapatite to the PHASES block (see Text S5 for code). We chose llnl.dat because it has been demonstrated to perform best in seawater-like ionic strength solutions.^29^

First, the aragonite and calcite saturation states of effluent wastewater ranged from 0.17 to 0.46 and 0.23 to 0.64, respectively, indicating consistent undersaturation. All samples from all depths were undersaturated with respect to aragonite (Figure 7C). As such, the dissolution of the primary KLL mineralogy is thermodynamically favorable. Calcite saturation states ranged from 0.2 (MN-1 15 m) to 1.5 (ME-1 6 m), with one outlier of 20.0 (MS-1 15 m), and did not significantly vary with depth (Figure 7C). Thus, secondary mineralization of calcite is possible at all depths if the kinetics are favorable. Finally, apatite-group minerals were strongly supersaturated in some samples at 3, 6, and 15 m.

Carbonate fluorapatite (CFA) is the most efficient means of P burial in marine sediments but forms exclusively within sediments as an authigenic mineral. It is thought that either a poorly crystalline Ca-rich precursor phase or HAP transforms to CFA during early marine diagenesis. Nearly every sample, excluding the MS-1 wells and MN-1 15 m in January 2023, was supersaturated with respect to HAP (Ω_HAP_ = 1.4–365.8; Figure 7, Figure S6), with the highest saturation states at 6 m. Precipitation of HAP should be favored in subsurface locations where mixing results in chemical desorption of P or carbonate mineral dissolution because of available Ca^2+^ and P. The relationship between excess [Ca^2+^] and removal efficiency of SRP within samples can be described by a logarithmic function (Figure 5C). Initially, significant SRP removal occurs without accumulation of excess Ca^2+^. Further removal is accompanied by the accumulation of Ca^2+^. This indicates that waters with a higher abundance of excess calcium, likely from calcium carbonate dissolution, more efficiently remove wastewater-derived SRP than waters without excess calcium (% excess [Ca^2+^] ∼0 in Figure 5C). In these shallow mixing zone waters where Ω_HAP_ ≫1, we interpret that SRP is removed as calcium phosphate minerals such as HAP. Note that there would be negligible removal of Ca^2+^ during HAP precipitation, given its orders of magnitude higher concentration than SRP. If this interpretation is correct, then the asymptotic approach to 100% SRP removal with time in Figure 5B could be reflecting the accumulation with time of excess Ca^2+^ from dissolution rather than true first-order kinetics of SRP removal.

### 4.4 Phosphorus Impacts on Nearshore Water Samples

There is significant uptake of phosphate onto the KLL prior to emerging to the nearshore waters. However, the concentrations of phosphate that can impact nearshore water quality and support macroalgal growth are small relative to the concentration of SRP in the wastewater effluent (<2 μM).^30,31^ Focusing on our Florida Bay adjacent wells MN-2 10′ and 20′, SRP concentrations were at or up to twice this level. Thus, it is likely that the discharge of groundwater into Florida Bay exceeds the 2 μM threshold established by ref (30). Sucralose was detected at all four nearshore sites, indicating anthropogenic inputs to each site. SRP was also detected in both the canal and mangrove sites in Boot Key Harbor, but no detectable concentrations of SRP were found at the northern locations in Florida Bay. This result is likely due to the fact that S1 and E1 sampling locations are more restricted from mixing with unaffected seawater than the samples taken from N1 and N2.

Although we found high rates of SRP removal through its interactions with KLL, wastewater-derived SRP is still reaching the nearshore waters: anthropogenic phosphorus may be contributing to the eutrophication of the nearshore waters and decline in water quality in the FKNMS. Quantification of the fraction of wastewater and concentrations of SRP found in groundwater along an effluent flowpath and in nearshore waters can help determine if shallow wastewater injection should be considered the functional equivalent of a direct point source contaminant and, in turn, inform decision making as required by the County of Maui vs Hawaii Wildlife Fund decision.^1^

## 5 Conclusions

Wastewater injection at the Area 3 Wastewater Treatment Facility in Marathon, Florida has changed the dynamics of groundwater flow and created an effluent plume that rises to the surface and interacts with the brackish near surface waters. Similar to earlier findings at the nearby Key Colony Beach injection facility, this system cannot be assumed to quantitatively remove anthropogenic phosphate in the subsurface, as has been true at smaller scale injection facilities. Significant depletion in wastewater-derived phosphorus was observed along the effluent flow path through the subsurface, on the short-term in the near proximity of injection and over longer time scales in wells with higher dissolved calcium concentrations than expected by ternary mixing of groundwater, wastewater effluent, and rainwater. We see no evidence that the phosphate uptake capacity is saturated even in the proximity of the injection well. At 3m, the effluent mixes with a shallow rainwater lens, which likely leads to calcium carbonate dissolution, phosphorus desorption, and calcium phosphate mineralization. Finally, environmentally significant concentrations of wastewater-derived phosphate are present in nearshore waters and could contribute to eutrophication.

The use of shallow injection as a disposal mechanism for treated wastewater should be re-evaluated at facilities with discharge capacities of this magnitude, and analytical and quantitative approaches such as those here should be used to determine whether wastewater injection can be considered the direct equivalent of a point source contaminant discharge.

## Supporting Information Available

The Supporting Information includes six supplemental figures referenced in text; detailed site description and additional background information, extended methods, dye tracer study, nearshore waters, and PHREEQc R code (PDF)
Additional Supporting Information in the form of an Excel file includes all data tables (XLSX)
