# Supplementary material for: The Efficiency of Phosphate Removal via Shallow Wastewater Injection into a Saline Carbonate Aquifer
Source: ACS ES T Water. 2024 Jul 26;4(8):3540–9. doi: 10.1021/acsestwater.4c00407 (PMC11320573; doi:10.1021/acsestwater.4c00407)
Supplement: Supplementary file 1 — ew4c00407_si_001.pdf [file ew4c00407_si_001.pdf]

Supplemental Information for

The efficiency of phosphate removal via shallow wastewater injection into a saline carbonate aquifer: Marathon, Florida

Kate Meyers[a], Megan Martin[a], Lee R. Kump[a], Miquela Ingalls[a]*

[a]Department of Geosciences, The Pennsylvania State University, PA 16802 USA

*email: ingalls@psu.edu

Contents

A. Supplemental Text

1. Detailed site description and additional background information 2
2. Extended methods 2
3. Dye tracer study 7
4. Nearshore waters 8
5. Phreeqc R code 9

B. Supplemental Figures 12

Supplemental Text

Supplemental Text 1. Detailed site description and additional background information

The Florida Keys National Marine Sanctuary (FKNMS) protects 9,800 km^2 of marine ecosystems and resources. The world's third largest coral barrier reef and over 1,700 islands are found within the sanctuary (Halley, 1997; Florida Keys National Marine Sanctuary and Protection Act, 1990). Each year, over five million tourists and local residents depend upon potable water provided by the Florida Aqueduct Authority (NOAA 2007). Wastewater has traditionally been disposed of by septic systems, cesspits, and primary treatment followed by shallow injection, but more recently, advanced wastewater treatment followed by shallow or deep injection has become the norm.

There has been a historical decline in water quality in Florida Bay and more recently an increase in observations of harmful algal blooms (HABs) in Florida waters (Accoroni et al., 2020; Boyer et al., 1999; Fourqurean & Robblee, 1999; Heil & Muni-Morgan, 2021). Although a great deal of interest has been focused on the quality of coastal waters in the Florida Keys, where tourism, which relies on the health of fragile coastal ecosystems, drives the local economy, these issues are non-unique to South Florida. As such, it is necessary to investigate the sources and impacts of nutrient loading in coastal regions broadly.

Supplemental Text 2. Extended methods.

Well installation. Five monitoring wells were installed in May 2021 within 300 m of the wastewater injection well and five in February 2022 by a commercial well-driller (JC Drilling, Miami, FL). (Figure 1, Table S1). At each location, wells are drilled to sampling depths of 3 m, 6 m, and 15 m, with sampling wells at 27 m at three sites (Table S1). At each well location, a ~15

cm well bore was drilled using a hollow-stem auger. The well cuttings were disposed of on-site. Each well was composed of a 2.5 cm diameter PVC pipe which extended to the surface. The bottom 1.5 m interval of the well had a 38 mm (0.015”) mesh-size slotted screen packed with coarse (6/20) sand at the depth of the sampling interval. The wells were backfilled with approximately 10 kg of 30/65 mesh silica sand, a layer of bentonite, then a mix of bentonite and Portland cement to the surface (Figure 2).

At the surface, a cement pad was constructed sloping away from the wells to prevent surface runoff from entering the wells. Lockable, metal protective casings were set into the cement. Inside the casing, wells were capped with standard PVC rubber tension caps. Following each drilling period, wells underwent an intense and prolonged purging period and then left to recover for longer than a month prior to geochemical sampling.

Well preparation. At least three well-volumes of water were purged from each well prior to sampling or field analysis. Depth to water measurements were taken before and after the purge. An Everbilt Utility Pump was used to purge the 15 and 27 m wells. A Masterflex peristaltic pump was used to collect the samples after flushing the Tygon tubing with well water for two minutes. The Masterflex peristaltic pump was used for purging and sampling the 3 and 6 m wells, as the utility pump stirred up sediment in the wells at shallower depths, increasing turbidity which can impact chemical measurements.

Sample collection. Some analyses require sample filtration prior to analysis while others do not. Water samples for analyses that do not require filtration were collected directly into high-density polyethylene (HDPE) bottles that were cleaned with phosphate free soap, acid washed in 6N HCl, and rinsed with sample three times prior to collection. Filtered samples were passed through two PALL GWV high-capacity groundwater sampling capsules in series, with filter sizes of 0.5 μm and 0.45 μm. Each monitoring well had its own filter array used throughout the two-year sampling interval. At the end of every sampling campaign, capsules were purged with DI water prior to storage. At the end of each sampling day, the Tygon tubing was flushed with at least three volumes of DI water. All samples were immediately preserved on wet ice in the field. Ten percent of samples were taken in duplicate for each analysis type.

Field analyses. A 1 L beaker was rinsed with groundwater at the depth of interest three times before collecting for conductivity, temperature, and pH measurements using an Orion Model 140 Portable Conductivity / Salinity / Temperature probe with stated accuracies of 0.2°C and conductivity/salinity to within 0.5%. Conductivity calibrations were made at the beginning of each sampling period. Dissolved sulfide was measured using the methylene blue assay in the field immediately after collection according to EPA method 376.2. Alkalinity samples were collected into overfilled 120 mL bottles and refrigerated until analysis within the same day using a Corning titrimetric alkalinity meter following EPA method 310.1.

Nearshore sampling. Samples were collected via kayak in 3-liter containers approximately 1 m below the surface. Sample containers were rinsed 5 times with nearshore water prior to collecting the final sample. Salinity and temperature data were measured at the time of sampling using a multiparameter sonde. Samples were transferred on ice to the lab. Glass bottles were filled directly from the sampling container for pharmaceutical analysis and aliquots for nutrient analysis were pumped out of the sampling container through a filtering array into acid washed bottles.

Nutrient Analysis. Samples were analyzed at the Florida International University (FIU) CAChE-Nutrient Analysis Core Facility. Total phosphorus (TP) samples were not filtered, and soluble reactive phosphorus (SRP) and dissolved organic carbon (DOC) samples were filtered through the 5 and 0.45 µm filter series. TP and SRP were analyzed following EPA method 365.1 and DOC followed method NU-062-1.8. SRP is the fraction of phosphorus that remains after filtration through a 0.45 µm pore size filter, effectively the measure of phosphate. SRP and DOC samples were frozen and TP samples were refrigerated until transport to FIU, at which point they were transported on ice for no more than two hours. All samples were delivered to FIU within 4 days of collection. In addition to the field replicates, 10% of the samples were selected for method duplication.

SRP samples remained frozen until the time of analysis and analyzed within 48 hours (CAChE-NACF SOP-004) once removed from the freezer. TP samples were maintained at 2-6°C for up to 28 days before analysis (CAChE-NACF SOP-008). The method detection limit (MDL) and practical quantification limit (PQL) in seawater and freshwater are listed in Table S3 in µM. Every analytical batch of 20 samples was preceded by the analysis of calibration curve standards in decreasing order of concentration and reagent blanks. Each group of no more than

10 samples were bracketed by continuing calibration verification standards and reagent blanks. If more than 20 samples were run, a set of quality control samples were required after every batch.

Pharmaceutical Analysis. Concentrations of specific pharmaceuticals and sucralose were analyzed at the Tallahassee Florida Department of Environmental Protection (FDEP) laboratory following the standard procedure EPA 8321B via high performance-liquid chromatography tandem mass spectrometry (HPLC/MS/MS) to track anthropogenic compounds derived from wastewater migration. The subset of compounds analyzed included acetaminophen, sucralose, carbamazepine, ibuprofen, and naproxen. Sucralose is an artificial sweetener present in the effluent and is not removed by AWT. This organic compound breaks down minimally in the environment and can be used as a conservative tracer representing wastewater effluent and therefore can distinguish the rainwater and wastewater endmembers that have similar impacts on salinity. Samples were filtered and collected in 500 mL amber glass bottles provided by the FDEP laboratory. They were stored in the refrigerator until being shipped on wet ice overnight to Tallahassee. The MDL and PQL for each component are listed in Table S4.

Ion Analysis. A geologic suite of anions and cations were measured at the PSU Laboratory for Isotopes and Metals in the Environment (LIME). Samples were collected in 60 mL HDPE bottles before being transferred to two 15 mL falcon tubes for shipping: one sample aliquot for anion analysis and one acidified with 2% nitric acid for cation analysis. Ion samples were transported at room temperature and analyzed within 3 weeks of each sampling campaign. Anions were measured via a Dionex ICS 2100 Ion Chromatography System (IC) and cations were measured via Thermo iCAP 7400 Inductively Coupled Plasma Emission Spectrometry (ICP-AES). The detection limits for each ion are listed in Table S5. Synthetic standards from High Purity Standards were used to calibrate the results. For anions, quality control (QC) standards containing a concentration of 1 and 10 ppm of each analyte were measured. For cations, the EPA 200.7 QC standard was diluted 1/100, 1/20, 1/10, and 1/4.

Supplemental Text 3. Dye tracer study

During the May 2022 sampling trip, the first phase of the dye tracer was initiated. Prior to dye injection, background fluorescence was measured at all well locations. On May 9th, 2022, 1.8 kg of fluorescein dye powder was mixed with 909 L of water in an opaque storage tank at the Area 3 Wastewater Treatment Facility. The dye was thoroughly mixed prior to being gravity fed from the storage tank into the effluent injection port of the wastewater treatment facility over approximately two and a half hours. The flow rate of effluent at the time of the dye study was 75 L/minute.

At the beginning of each sampling day an AquaFluor Handheld Fluorometer was calibrated with a blank, a 10 ppb and 400 ppb standard prior to running any field samples. All wells were purged following the same methods as 2.1.2. During the purge, water was collected in buckets until fluorescence measurements were taken. If the groundwater measured less than

1 ppb it was dumped on pavement near the well and assumed to have minimal interaction with the groundwater and the experiment. If the water was measured greater than 1 ppb, it was collected in a storage tank for the duration of the study.

MW-0 wells were purged and measured for fluorescence 6 times over the course of 3 hours after the initial dye injection to observe the arrival of the dye pulse at the most proximal wells. Fluorescence was measured twice per day at the MW-0 wells on the second and third day after dye injection. On the third and fourth day of the study, the nearest 4 well locations were also sampled and immediately analyzed for fluorescence, ME-1, MW-2, MN-1, and MS-1. Sampling occurred daily for the first 10 days of the study at 3 of the 5 central wells. After this point sampling slowed to 3-4 days per week, focusing on the central wells with occasional sampling of the outermost well locations (ME-2, MN-2, MW-3, MS-2). As the dye arrived at different well locations sampling became more frequent as needed to capture the full peak of arrival (Table S2).

Because of concerns that sufficient dye was not injected to detect fluorescein at the most distal wells, a second more concentrated pulse of dye was injected in June 2022. The amount of dye injected during the initial experiment was determined not to be concentrated enough to be detectable at the outermost wells after groundwater dilution in the plume travel path. 11.8 kg of fluorescein dye powder dissolved into 909 L of water was pumped into the wastewater effluent injection well. During this injection, a pool pump was used to feed the dye into the well at a more consistent rate over the course of 30 minutes. Samples were analyzed at two to three well locations per day for the first week after this second injection and then steadily decreased in frequency over the course of the study as peak arrivals were detected.

Peak arrival of the dye patch was detected at the well cluster within the confines of the Area 3 Treatment Facility within 24 to 72 hours of injection (Figs. S3-4), and at ME-1 and MN-1 within one to two weeks. Due to the complex hydrology including potentially stagnant water within a karst cavity at MS-1, we were not able to detect a fluorescence peak at MS-1. Further,

we suspect that the ~1-2 ppb levels of fluorescein detected at MS-1 could be attributed to interferences in the fluorescence measurements due to turbidity of the samples. No peak was detected as of January 2024 or was missed in between sampling intervals at ME-2 and MW-3.

Supplemental Text 4. Nearshore waters

The highest concentration of sucralose in the nearshore waters (370 ng/L) was found at E1 in largely restricted waters of a canal. The next highest concentration of sucralose was found at S1 located along the mangroves ~150 m south of MS-3 in the partially restricted waters of Boot Key Harbor. Finally, the lowest concentrations of sucralose of the nearshore samples were found on the Florida Bay side of Marathon at N1, these sampling locations were located ~20 and ~25 m north of the northernmost piezometer nest MN-2.

Supplemental Text 5. Phreeqc R code.

```
remove(list= ls()) # Clear everything  # Clear the workspace from the previous run

library(readr)
data <-as.data.frame(read_csv("Marathonphreeqc.csv")) # Read in your data as a .csv
spreadsheet.
print(typeof(data))

library(phreeqc)                    # Load the PHREEQC library
phrLoadDatabaseString(llnl.dat)      # Use the pitzer database

# Assign your variables following the format: data$columnname

temp <- data$T_C
pH <- data$pH
Ca <- data$Ca_mM
Na <- data$Na_mM
K  <- data$K_mM
Mg <- data$Mg_mM
Cl <- data$Cl_mM
```

```
Sr <- data$Sr_mM
SO4 <- data$SO4_mM
Fe <- data$Fe_mM
Si <- data$Si_mM
Br <- data$Br_mM
P <- data$P_mM
Alkalinity <- data$TA

n = length(pH) # this can be whichever column, it is just telling the loop the number of rows run

# Create empty vectors for the desired output variables

si_Calcite <- rep(0, n)
si_Aragonite <- rep(0,n)
si_Hydroxyapatite <- rep(0,n)
omega_Calcite <- rep(0, n)
omega_Aragonite <- rep(0,n)
omega_Hydroxyapatite <- rep(0,n)
CO2 <- rep(0, n)
HCO3 <- rep(0, n)
CO3_free <- rep(0, n)
DIC <- rep(0, n)

################################## PHREEQC input
####################################

for (loop in 1:n){
  step = loop
  input <- c(
    'PHASES           ',
    'Hydroxyapatite   ',
    '    Ca5(OH)(PO4)3 +4.0000 H+  =  + 1.0000 H2O + 3.0000 HPO4-- + 5.0000 Ca++ ',
    '    log_k           -3.0746 ',
    '       -delta_H         -191.982         kJ/mol # Calculated enthalpy of reaction ',
    ' #    Enthalpy of formation:-6685.52 kJ/mol ',
    '    -analytic -8.5221e+002 -2.9430e-001 2.8125e+004 3.4044e+002 4.3911e+002 ',
    ' #       -Range:  0-300 ',
    'SOLUTION           ',
    paste('temp  ',    temp[step], sep = ' '),
    paste('Alkalinity  ',   Alkalinity[step], sep = ' '),
    'units       mmol/kgw',
    paste('Ca  ',   Ca[step], sep = ' '),
    paste('CO2  ',    CO2[step], sep = ' '),
    paste('HCO3-  ',    HCO3[step], sep = ' '),
```

```
    paste('CO3-2  ',    CO3_free[step], sep = ' '),
    paste('Na  ',    Na[step], sep = ' '),
    paste('pH  ',    pH[step], sep = ' '),
    paste('K  ',    K[step], sep = ' '),
    paste('Mg  ',    Mg[step], sep = ' '),
    paste('Cl  ',    Cl[step], sep = ' '),
    paste('Fe  ',    Fe[step], sep = ' '),
    paste('Br  ',    Br[step], sep = ' '),
    paste('Si  ',    Si[step], sep = ' '),
    paste('Sr  ',    Sr[step], sep = ' '),
    paste('P  ',    P[step], sep = ' '),
    paste('S(+6)  ',    SO4[step], sep = ' '),

    'SELECTED_OUTPUT            ',
    '      -si Calcite        ',
    '      -si Aragonite       ',
    '      -si Hydroxyapatite    ',
    '      -molalities CO2  HCO3- Alkalinity CO3-2 C(4)')

  phrRunString(input)                    # Run the input string

  output <- phrGetSelectedOutput()       # and save the results in 'output'

  si_Calcite[step] <- output$n1$si_Calcite
  si_Aragonite[step] <- output$n1$si_Aragonite
  si_Hydroxyapatite[step] <- output$n1$si_Hydroxyapatite
  omega_Calcite[step] <- 10^output$n1$si_Calcite
  omega_Aragonite[step] <- 10^output$n1$si_Aragonite
  omega_Hydroxyapatite[step] <- 10^output$n1$si_Hydroxyapatite
  CO2[step]      <-   output$n1$m_CO2.mol.kgw.
  HCO3[step]     <-   output$n1$m_HCO3..mol.kgw.
  CO3_free[step] <-   output$n1$m_CO3.2.mol.kgw.
  DIC[step] < output$n1$m_CO2.mol.kgw. + output$n1$m_HCO3..mol.kgw. +
output$n1$m_CO3.2.mol.kgw.
} # Matching bracket to close loop

################################ PHREEQC output
###################################

output_df <- data.frame(CO2, HCO3, CO3_free, DIC, omega_Calcite, omega_Aragonite,
omega_Hydroxyapatite) # Create a dataframe with the desired output variables

Output_df # This prints the new output dataframe in R
```

write.csv(output_df, "saturation states llnl.csv") # This saves the dataframe as a comma separated list in your current directory. Title the new data frame as whatever you want

Supplemental Figures

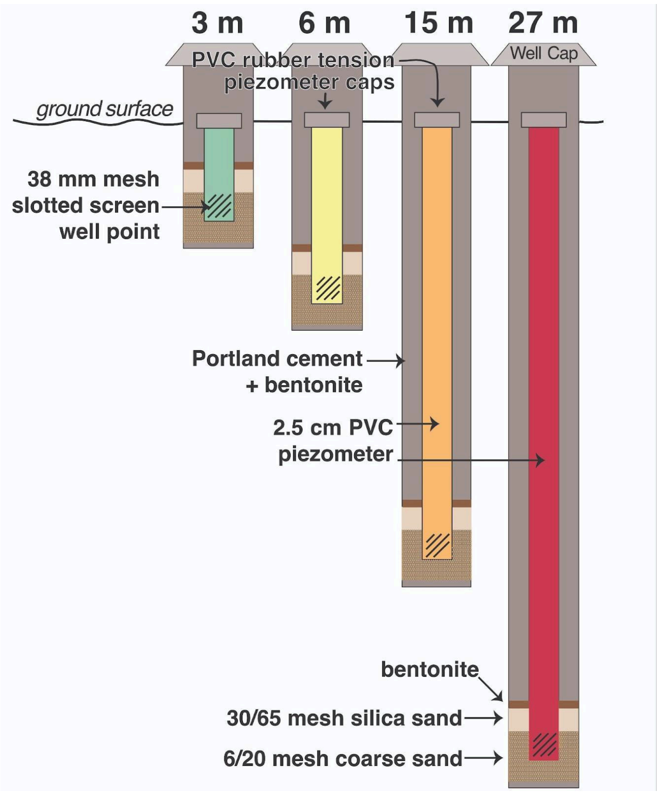

Figure S1. Schematic of piezometer nest design.

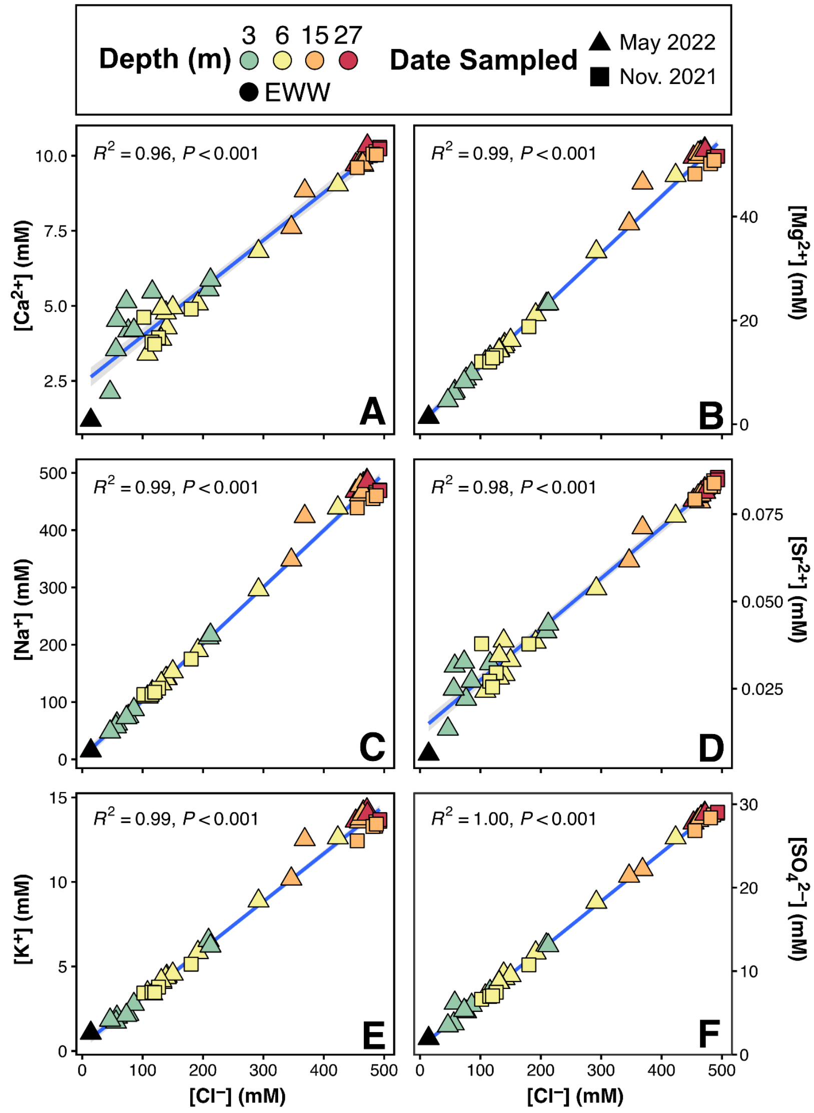

Figure S2. Concentrations of major ions vs chlorine concentration for the November 2021 and May 2022 samples. The confidence interval on the linear regression is 95%.

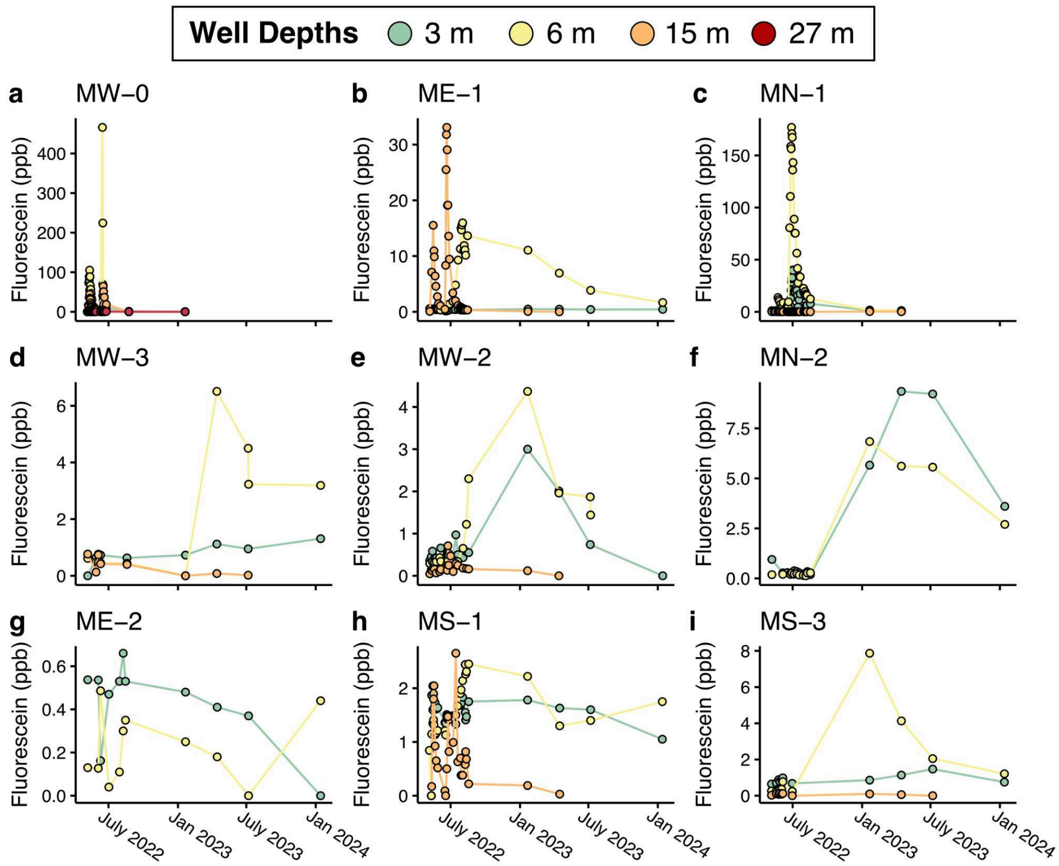

Figure S3. Fluorescein concentration curves through the duration of the dye tracer experiment, June 2022 to January 2024, for each well cluster. Peak arrival was not detected at all locations.

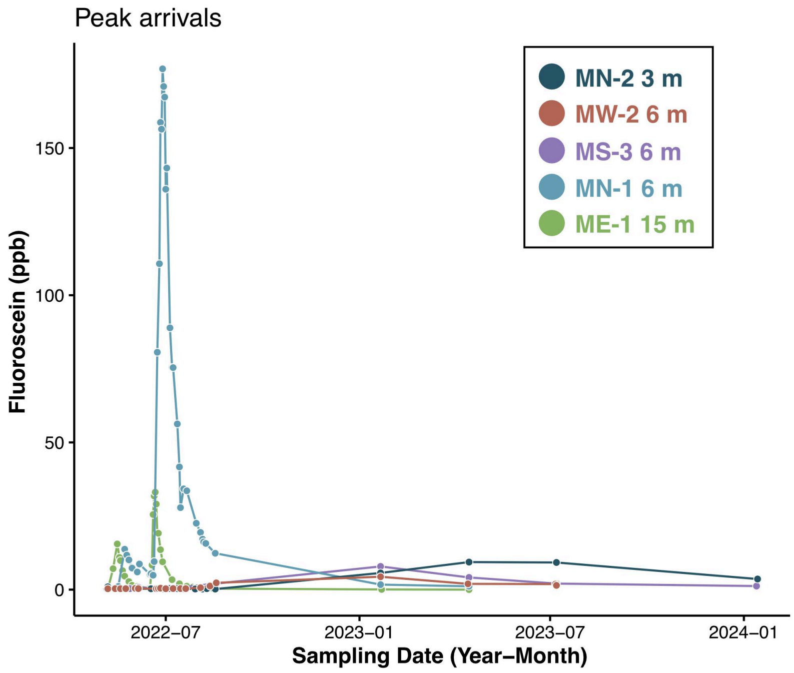

Figure S4. Peak arrival of fluorescein dye pulse at the well clusters and depths where peak arrival was detected. The fastest arrival was at ME-1 15 m depth, but the highest concentration of a peak arrival was at MN-1 6 m depth.

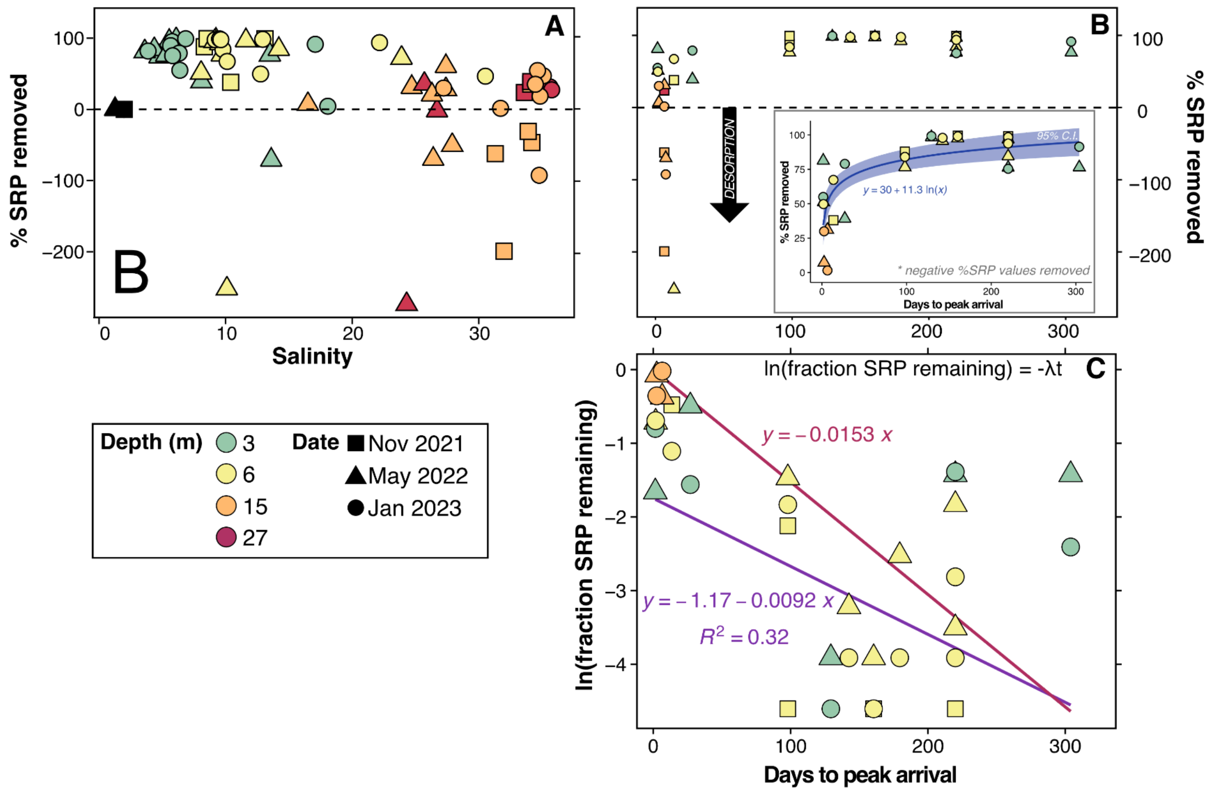

Figure S5. The relationship between SRP removal efficiency and (A) salinity and (B, C) transport time in the subsurface. (A) The highest rates of SRP desorption were observed in the highest salinity samples. (B) A rapid SRP removal phase was observed followed by a slower removal phase towards 100% SRP removal efficiency. (C) The rapid removal pathway yielded an SRP e-folding time of 12 days (purple) and the slower removal pathway yielded an SRP e-folding time of 66 days (red).

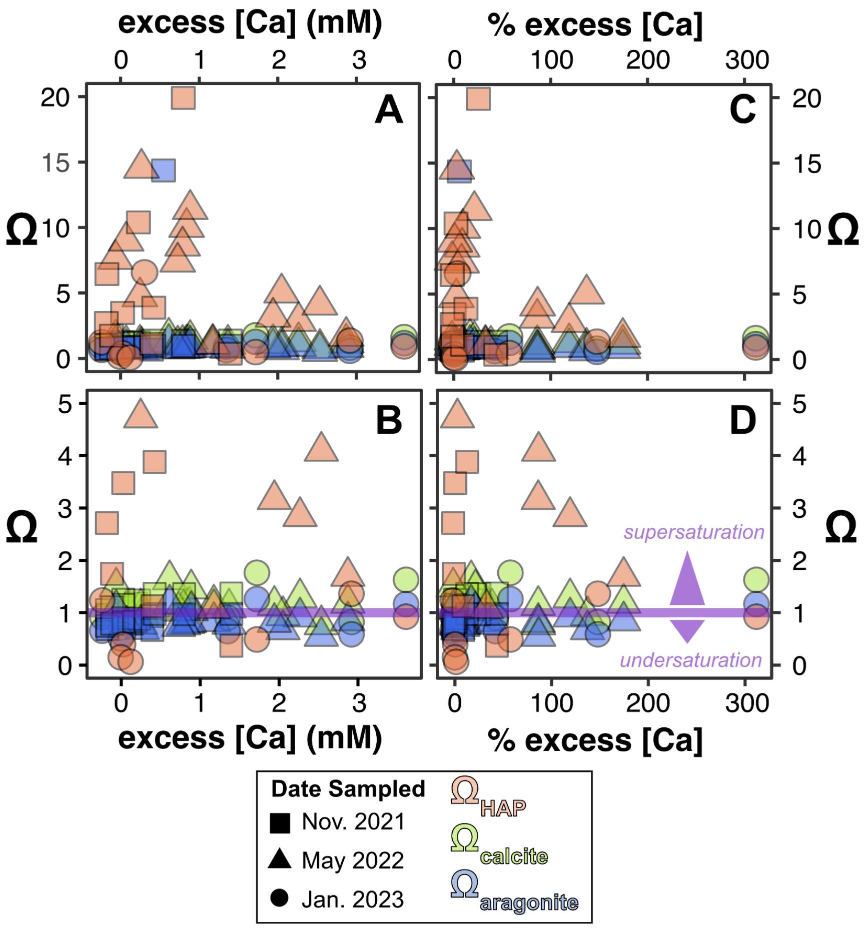

Figure S6. Hydroxyapatite, calcite, and aragonite saturation states versus excess calcium in solution.
